# Associations Between Diabetic Neuropathy and Balance Impairments in Patients with Type 2 Diabetes: A Cross-Sectional Study

**DOI:** 10.3390/jcm14238323

**Published:** 2025-11-23

**Authors:** Bianca Iliescu, Andreea Herascu, Laura Gaita, Vlad-Florian Avram, Bogdan Timar

**Affiliations:** 1 Doctoral School of Medicine, “Victor Babes” University of Medicine and Pharmacy, 300041 Timisoara, Romania; bianca.iliescu@umft.ro; 2 Sebes Municipal Hospital, 515800 Sebes, Romania; 3 Centre for Molecular Research in Nephrology and Vascular Disease, “Victor Babes” University of Medicine and Pharmacy, 300041 Timisoara, Romania; gaita.laura@umft.ro (L.G.); avram.vlad@umft.ro (V.-F.A.); bogdan.timar@umft.ro (B.T.); 4 Department of Diabetes, “Pius Brinzeu” Emergency Hospital, 300736 Timisoara, Romania; 5 Second Department of Internal Medicine, “Victor Babes” University of Medicine and Pharmacy, 300041 Timisoara, Romania

**Keywords:** type 2 diabetes, diabetic neuropathy, balance impairment, gait, accidental falls

## Abstract

**Background:** Diabetic neuropathy (DN) may impair balance and gait, increasing the risk of falls in elderly patients with type 2 diabetes mellitus (T2DM). This study aimed to evaluate whether the presence and severity of DN are associated with balance and mobility impairment as well as with increased fear of falling in patients with T2DM. **Methods**: A total of 124 adults with T2DM underwent neuropathy assessment with the Michigan Neuropathy Screening Instrument (MNSI) and sudomotor testing (SUDOSCAN). Balance and fall risk were evaluated using the Berg Balance Scale (BBS), Timed Up and Go (TUG), Falls Efficacy Scale—International (FES-I), and Fear of Falling Questionnaire—Revised (FFQ-R). Comparison of parameters between patients with vs. without DN, correlations, and multivariable linear regressions (DN components as predictors) were performed. **Results**: Compared with those without DN, participants with DN had higher FES-I (31.0 vs. 21.0) and FFQ-R (56.0 vs. 42.0) scores and lower BBS (42.0 vs. 46.0). TUG did not differ significantly (11.8 vs. 11.25 s). In multivariable models, higher MNSI questionnaire and objective scores independently predicted lower BBS (*β* = −0.74 and −1.1, respectively) while only the MNSI questionnaire predicted higher TUG (*β* = 0.43). For fear of falling, the MNSI questionnaire predicted higher FES-I (*β* = 1.66) and both MNSI components predicted higher FFQ-R (*β* = 2.31 and 1.7, respectively). Leg SUDOSCAN values were not associated with BBS, TUG, FES-I, or FFQ-R. **Conclusions**: DN is associated with impaired balance and greater fear of falling. Neuropathy burden, particularly patient-reported symptoms, relates to worse performance and confidence, whereas sudomotor impairment alone does not.

## 1. Introduction

Diabetic neuropathy (DN) is one of the most prevalent complications in patients with diabetes [1]. Studies have shown large heterogeneity in the prevalence of diabetic neuropathy, ranging from 20 to 30% in cohorts of patients with newly diagnosed diabetes, up to more than 80% in patients with long-standing diabetes duration, leading to an important increase in the overall risk as well as to significant impairments of the quality of life in these patients. At the same time, DN seems to be more prevalent in patients with type 2 diabetes mellitus (T2DM) compared to those with type 1 diabetes mellitus (T1DM) [2].

One of the risk factors for developing T2DM is age. It has been observed that fasting plasma glucose rises by about 2 mg/dL per decade during normal aging, increasing the risk of diabetes in elderly persons [3]. The prevalence of diabetic neuropathy has also been shown to increase with age, rising from 11.9% among individuals aged <40 years to over 50% in those older than 70 years [4].

Falls are a major health concern for elderly persons, particularly for those with T2DM, in which healing after trauma is impaired due to the intrinsic consequences of diabetes [5]. Falls are common in this elderly population and can lead to serious injuries, their consequences being emphasized in persons with T2DM [6]. Additionally, these individuals must cope with condition-specific challenges, including neuropathy, retinopathy, proprioceptive changes, cognitive impairment, and hypotension, as well as age-related declines in balance, muscle strength, and walking ability [5].

Studies have shown that the risk of sarcopenia is 1.5–2 times higher in patients with T2DM compared to those without the disease [7]. Furthermore, sarcopenic obesity significantly increases the risk of T2DM due to insulin resistance and impaired glucose metabolism [8]. Sarcopenia may increase the risk of falls in older adults by impairing postural control and exacerbating fear of falling [9].

DN elevates the risk of falls and adversely affects stride kinematics and postural stability [10]. Neuropathy may cause balance disorders such as autonomic (resting tachycardia, orthostatic hypotension, and impaired adaptation to changes in lighting), motor (muscle weakness and impairment of movements), and sensory (reduced proprioception with impaired postural adaptation) disorders [11].

Although several studies have documented that DN impairs gait, balance, and activities of daily living and increases fall risk, most have not comprehensively assessed both objective performance measures and psychological fear-of-falling constructs in the same cohort of elderly patients with T2DM. Furthermore, previous work has focused mainly on large-fiber peripheral neuropathy, with very limited data on the role of small-fiber and autonomic (sudomotor) dysfunction in balance control. To our knowledge, no previous study has simultaneously combined a standard clinical neuropathy score (MNSI), sudomotor function evaluated by SUDOSCAN, and both performance-based and fear of falling instruments in this population.

Considering all of these premises, this study aims to assess the extent to which the presence and severity of neuropathy are associated with the occurrence and severity of balance and gait impairment in patients with T2DM.

## 2. Materials and Methods

### 2.1. Study Design and Patients

In this cross-sectional, monocenter, observational, non-interventional study, 124 patients with T2DM, aged higher than 60 years old, were included according to a consecutive-case, population-based enrollment principle. Patient visits took place between September 2023 and April 2024 and data were entered in the database between September 2023 and December 2024. All participants had to have a diabetes duration longer than 6 months. The rationale for the age inclusion limit and diabetes duration was to increase the sensitivity of the study, considering that elderly patients are more prone to developing balance impairment and a duration longer than 6 months was considered the minimum clinical exposure to diabetes to be able to evaluate its impact on balance parameters. The following were exclusion criteria: patients unwilling to provide written informed consent to participate in the study or unwilling to perform the dynamic balance tests, patients unable to provide detailed information regarding their medical history, and patients with a history of non-diabetic neuropathies, major cardiovascular events in the 3 months prior to the enrollment visit, or any other medical conditions which, in the investigator’s opinion might bias the study’s results. The study protocol and patient’s informed consent form were approved prior the start of the study by the Ethics Committee of the “Victor Babeș” University of Medicine and Pharmacy, Timisoara, Romania (Approval Number 27/05.07.2023). All patients were enrolled in the outpatient clinic of the Brad Municipal Hospital, Hunedoara County, Romania.

Patients’ baseline characteristics are presented in Table 1.

### 2.2. Neuropathy Assessment

The presence of diabetic neuropathy was diagnosed both by using the Michigan Neuropathy Screening Instrument (MNSI), as well as by measuring the sudomotor dysfunction of the skin, using the SUDOSCAN TM device. MNSI is a clinically validated instrument, including for the Romanian population, and consists of two major components: a questionnaire with 15 items, in which the healthcare professional assesses the subjective neuropathy symptoms reported by the patient, and an objective, scored evaluation of the patient’s feet, performed by the healthcare personnel. To avoid between-observer and methodological biases, the tests were performed by the same operator in the same examination setting and conditions. A score higher or equal to 7 points on the questionnaire or a score higher or equal to 2 on the objective evaluation was considered a positive test for the diagnosis of neuropathy. A higher MNSI score is associated with more signs and symptoms associated with neuropathy.

SUDOSCAN ^TM^ (Impeto Medical, Paris, France) is an objective, clinically validated test for diagnosing sudomotor impairment in diabetic neuropathy. SUDOSCAN measures the electrochemical conductance of sweat in both feet and hands. A lower conductance is associated with more severe impairment of the sudomotor function, affected by neuropathy. A more severe impairment of feet vs. hands is suggestive for diabetic neuropathy, while an increased asymmetry between feet or between hands is highly suggestive for other causes of neuropathy than diabetes-related etiology. A score between 60 and 100 µS is considered normal sudomotor function, between 40 and 60 µS is considered moderately reduced sudomotor function, and between 0 and 40 µS is considered severely reduced sudomotor function. In this study, SUDOSCAN assessments were performed according to the manufacturer’s recommendations, with the patients standing barefoot on the electrodes in a quiet examination room. The device underwent regular calibration and built-in quality-control checks as recommended by the manufacturer, and electrode plates were cleaned and dried between measurements. To avoid the possibility of between-device or inter-operator biases, all measurements were performed by the same operator, using the same device. SUDOSCAN has previously demonstrated good short-term reproducibility and test–retest reliability in patients with diabetes, supporting the use of single measurements per limb in clinical research.

### 2.3. Balance Impairment Assessment

To diagnose balance impairment, the following instruments were used: Falls Efficacy Scale—Internation (FES-I), Fear of Falling Questionnaire—Revised (FFQ-R), Berg Balance Scale (BBS), and Timed Up and Go test (TUG). The FES-I is a self-report questionnaire designed to assess the concern about falling during the performance of various daily activities, both basic activities as well as more demanding ones. It includes 16 items rated on a 4-point Likert scale (where 1 means not at all concerned and 4 means very concerned), with higher scores indicating greater fear of falling and reduced confidence in balance. FFQ-R is a psychometric tool that evaluates the intensity and impact of fear of falling in elderly adults. It measures both emotional concern as well as behavioral adaptations related to balance loss, providing a multidimensional assessment of how fear of falling affects daily functioning. A higher score in the FFQ-R instrument is considered to be associated with a more severe balance impairment The BBS is a performance-based measure, consisting of 14 functional tasks (such as standing up, turning, reaching, and single-leg stance) that assess static and dynamic balance. Each item is scored on a 5-point scale (between 0 and 4), with a maximum score of 56. A lower score in BBS is associated with more severe balance impairment. The TUG is a widely used clinical test of mobility and balance. It measures the time (in seconds) an individual requires to rise from a standard chair, walk 3 m, return and sit down again. Shorter times at TUG are associated with better functional mobility, while longer times are associated with impaired balance and increased risk of falls.

### 2.4. Clinical, Anthropometric, and Laboratory Assessments

In all patients, complete and structured anamnesis (including complete medical and therapeutic history, and diabetes history) was performed by the same evaluator and data were recorded in a standardized collection form. Patient height and weight were measured in a morning fasting state using the same standardized and calibrated stadiometer and body weight scale. Laboratory tests were performed at the same laboratory, using the same method and calibration method. The following laboratory tests were performed in all patients: hemoglobin A1c (HbA1c), total cholesterol, low-density lipoprotein cholesterol (LDLc), high-density lipoprotein cholesterol (HDLc), triglycerides, aspartate aminotransferase (AST), alanine aminotransferase (ALT), calcium, magnesium, fibrinogen, high-sensitivity C-reactive protein (hsCRP), complete blood count, and creatinine.

### 2.5. Statistical Analysis

Data were collected and analyzed using MedCalc Statistical Software version 23.2.8 (MedCalc Software Ltd., Ostend, Belgium; https://www.medcalc.org; 2025).

The sample size was estimated before the start of the data collection to achieve a confidence level of 95% (1 − *α* = 0.05) in parallel with a statistical power of 80% (1 − *β* = 0.2). The calculus was performed based on the hypothesis that on the primary objective, the presence of diabetic neuropathy will decrease the median BBS score by at least 5 points, in parallel with an interquartile distance lower than 10 points versus patients without neuropathy. This estimation provided a sample size of 120 patients needed for the primary objective of the study. Considering possible drop-offs from the study, 124 patients were enrolled, and all patients completed the study.

All continuous variables collected in the study were tested for normality before analysis, using the Shapiro–Wilk normality test (nonparametric distribution was assumed for a *p*-value lower than 0.05 at the normality test).

Continuous variables with Gaussian distribution are presented as mean ± standard deviation and the statistical significance of differences between groups was assessed using unpaired *t*-student’s test. Continuous variables with non-parametric distribution are presented as medians and [interquartile range]; the statistical significance of difference in medians between groups was evaluated using the Mann–Whitney U test. Categorical variables are presented as absolute frequencies and (percentage of the total); the statistical significance of the differences between groups was assessed using the chi-squared test.

The strength of the association between two continuous variables was evaluated with Spearman’s correlation coefficient and its statistical significance was calculated using the *t*-value distribution technique.

To distinguish between independent interactions versus confounding factors, multiple regression analysis models were built.

In this study, the threshold for statistical significance was considered to be a *p*-value lower than 0.05.

## 3. Results

**Figure 1:**
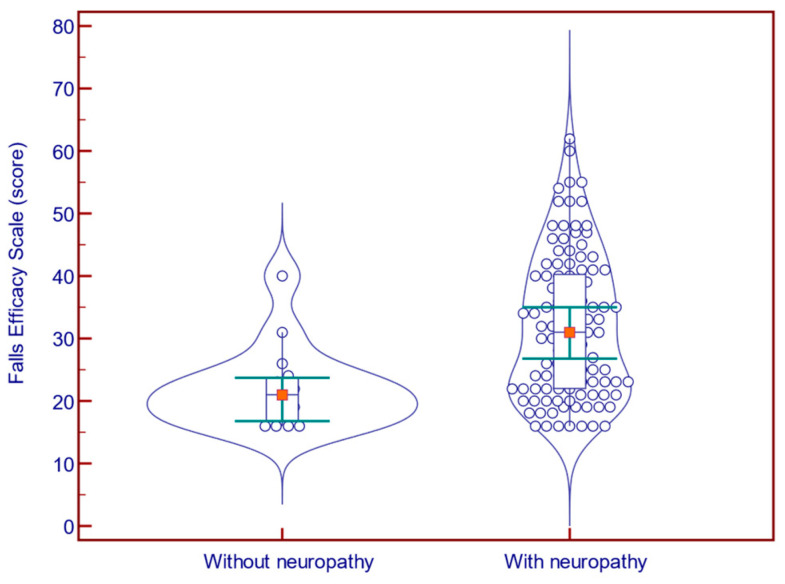
Falls Efficacy Scale comparison in patients with (n = 109) vs. without (n = 15) neuropathy.

**Figure 2:**
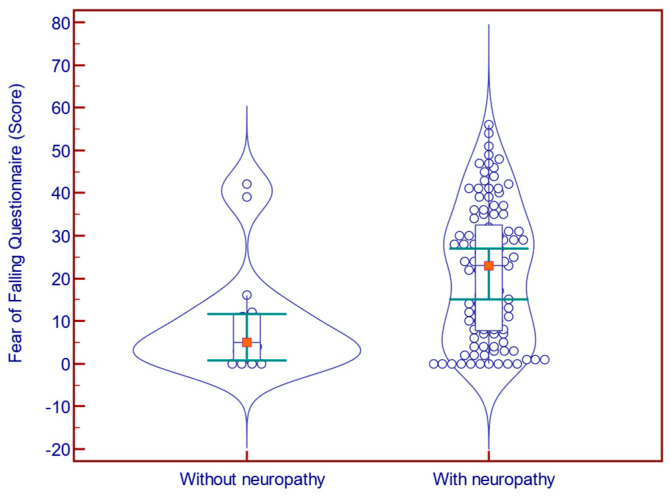
Fear of Falling Questionnaire scores in patients with (n = 109) vs. without (n = 15) neuropathy.

**Figure 3:**
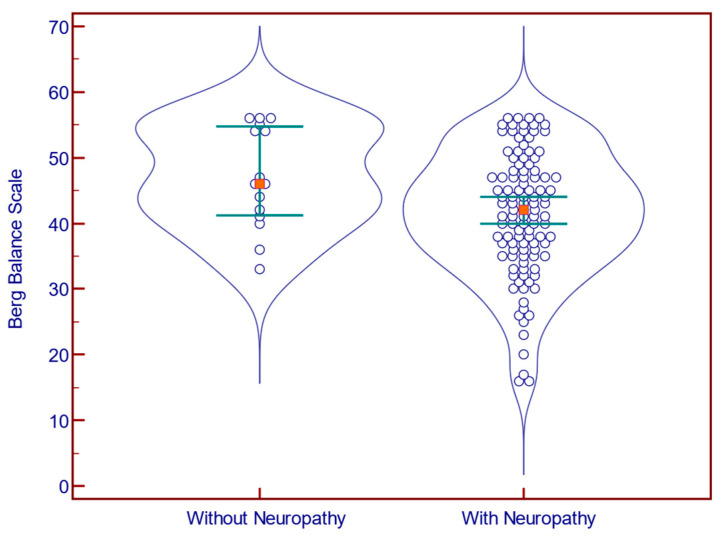
Berg Balance Scale scores in patients with (n = 109) vs. without (n = 15) neuropathy.

Of the 124 participants, 109 had met the MNSI criteria for DN. The presence of neuropathy was associated with a significantly increased FES-I score (31.0 vs. 21.0 points; *p* = 0.001; Mann–Whitney U test; Figure 1) and FFQ-R score (23.0 vs. 5.0; *p* = 0.006; Mann–Whitney U test; Figure 2), and with a significantly decreased BBS score (42.0 vs. 46.0 points; *p* = 0.029; Mann–Whitney U test; Figure 3).

No significant associations between the presence of neuropathy and TUG test were observed (11.8 vs. 11.25 s; *p* = 0.699; Mann–Whitney U test). The comparison of balance parameters between patients with vs. without neuropathy is presented in Table 2.

**Figure 4:**
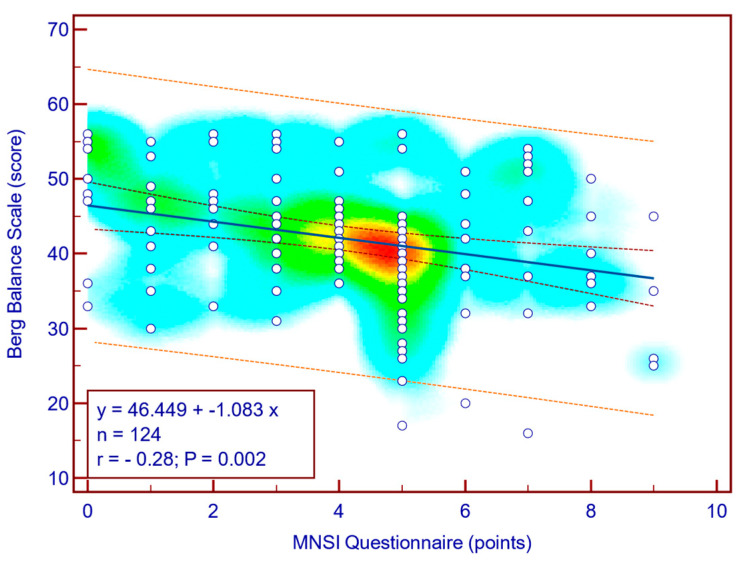
Correlation between BBS score and MNSI questionnaire score (n = 124).

**Figure 5:**
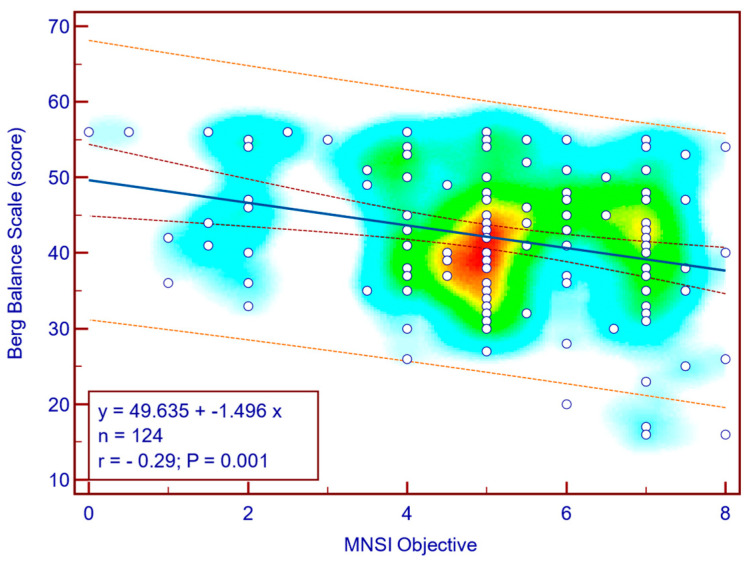
Correlation between BBS score and MNSI objective score (n = 124).

The BBS score was moderately, significantly, reverse correlated with both MNSI questionnaire scores (Spearman’s r = −0.28; *p* = 0.002; Figure 4) as well as with MNSI objective evaluation scores (Spearman’s r = −0.29; *p* = 0.001; Figure 5), suggesting that more severe neuropathy symptoms are associated with decreased balance in patients with T2DM.

**Figure 6:**
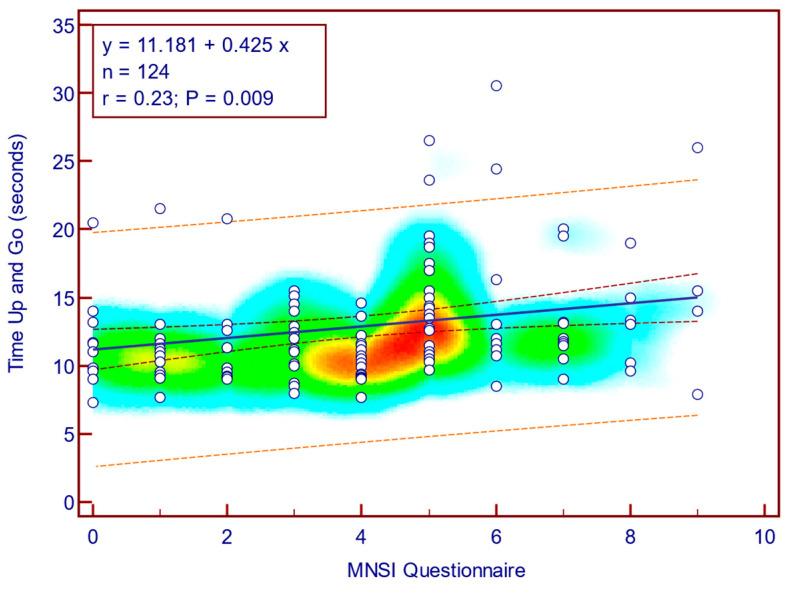
Correlation between TUG and MNSI questionnaire score (n = 124).

**Figure 7:**
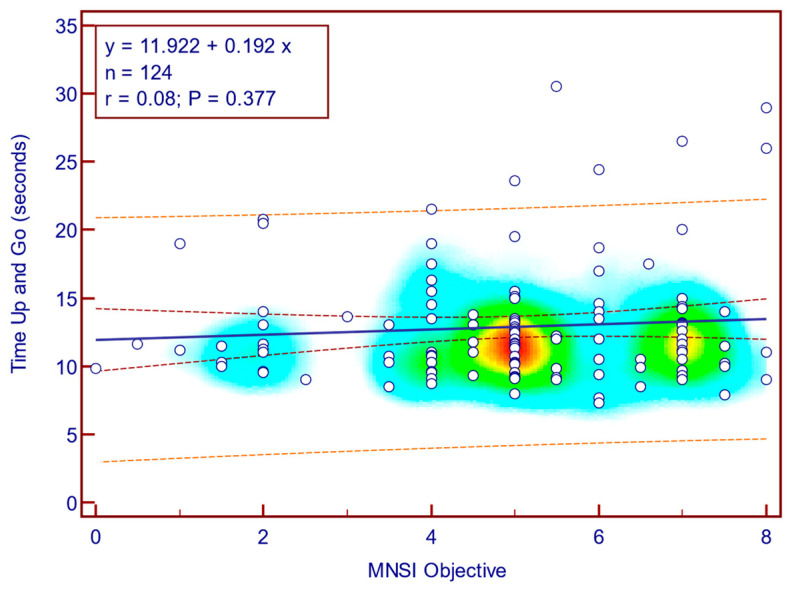
Correlation between TUG and MNSI objective score (n = 124).

The TUG score was moderately, significantly, reverse correlated with MNSI questionnaire scores (Spearman’s r = 0.23; *p* = 0.009; Figure 6), whereas its correlation with MNSI objective evaluation scores was weak and not statistically significant (Spearman’s r = 0.08; *p* = 0.377; Figure 7). This suggests that self-reported neuropathy symptoms are more closely related to mobility limitations than the examination component alone.

**Figure 8:**
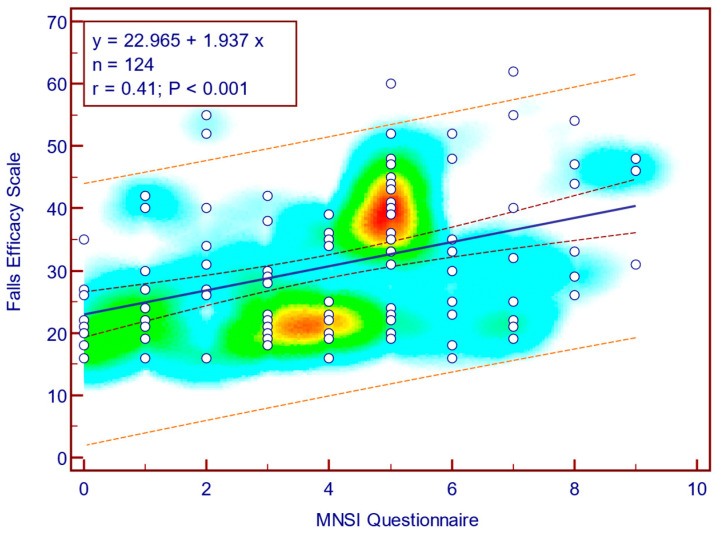
Correlation between FES-I score and MNSI questionnaire score (n = 124).

**Figure 9:**
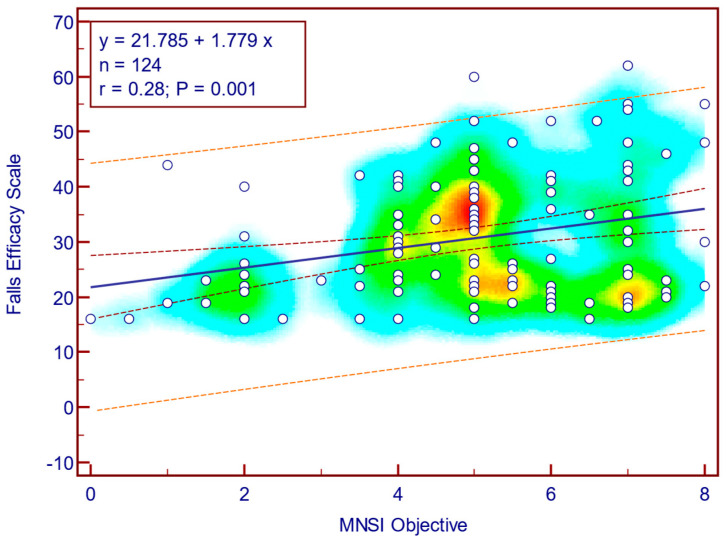
Correlation between FES-I score and MNSI objective score (n = 124).

The FES-I score was moderately, significantly, and positively correlated with both MNSI questionnaire scores (Spearman’s r = 0.41; *p* = <0.001; Figure 8) as well as with MNSI objective evaluation scores (Spearman’s r = 0.28; *p* = 0.001; Figure 9), suggesting that more severe neuropathy symptoms are associated with fear of falling in patients with T2DM.

**Figure 10:**
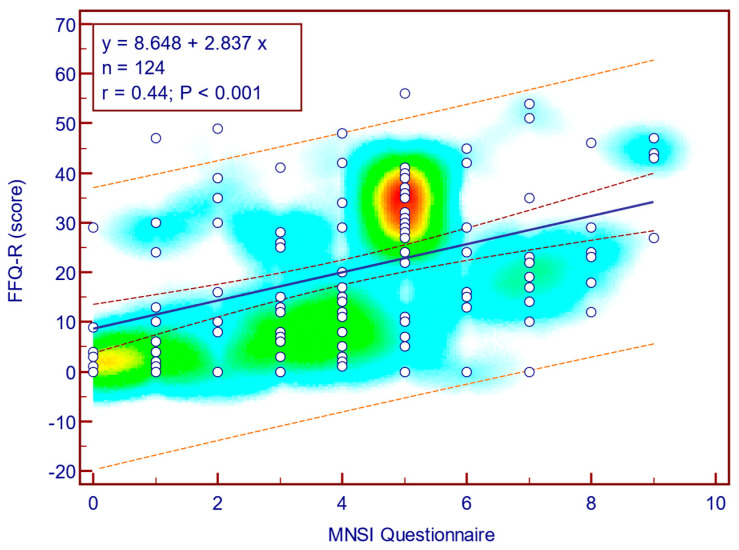
Correlation between FFQ-R score and MNSI questionnaire score (n = 124).

**Figure 11:**
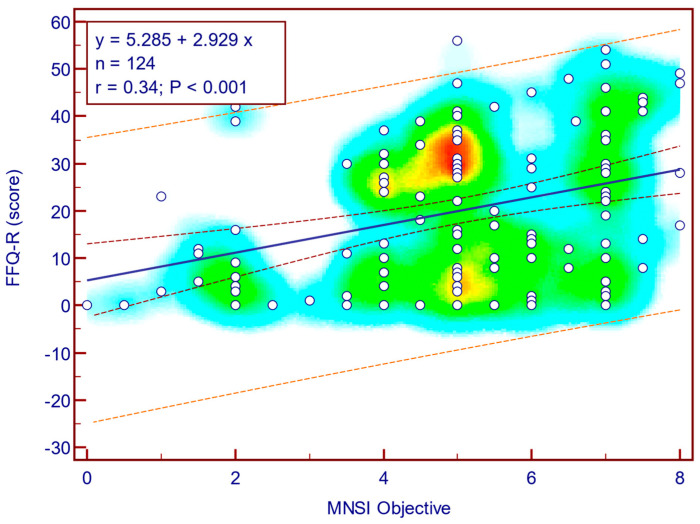
Correlation between FFQ-R score and MNSI objective score (n = 124).

The FFQ-R score was moderately, significantly, and positively correlated with both MNSI questionnaire scores (Spearman’s r = 0.44; *p* = <0.001; Figure 10) as well as with MNSI objective evaluation scores (Spearman’s r = 0.34; *p* < 0.001; Figure 11), suggesting that more severe neuropathy symptoms are associated with fear of falling in patients with T2DM.

**Figure 12:**
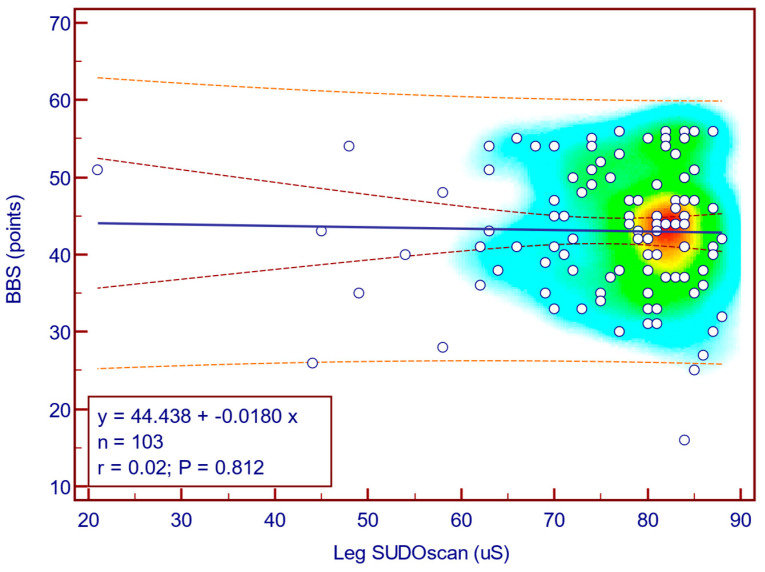
Correlation between BBS score and leg SUDOSCAN values.

The BBS score was weakly and not statistically significantly correlated with leg SUDOSCAN values (Spearman’s r = 0.02; *p* = 0.812; Figure 12), suggesting that there is no association between the severity of sudomotor dysfunction and functional balance in patients with T2DM.

**Figure 13:**
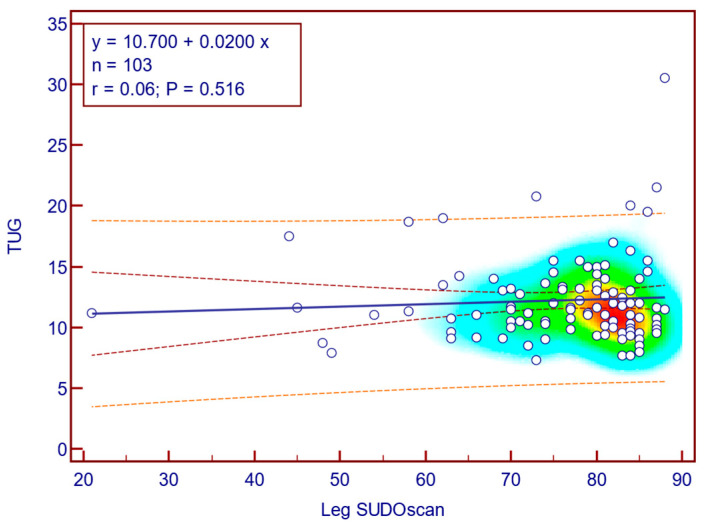
Correlation between TUG score and leg SUDOSCAN values.

The TUG score was weakly and not statistically significantly correlated with leg SUDO scan values (Spearman’s r = 0.06; *p* = 0.516; Figure 13), suggesting that there is no association between the severity of sudomotor dysfunction and fall risk in patients with T2DM.

**Figure 14:**
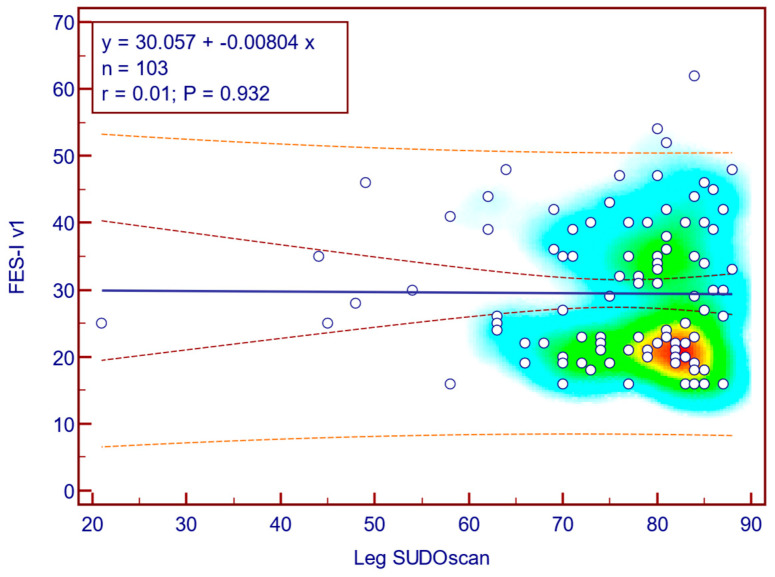
Correlation between FES-I v1 score and leg SUDOSCAN values.

The FES-I v1 score was weakly and not statistically significantly correlated with leg SUDOSCAN values (Spearman’s r = 0.01; *p* = 0.932; Figure 14), suggesting that there is no association between the severity of sudomotor dysfunction and fear of falling in patients with T2DM.

**Figure 15:**
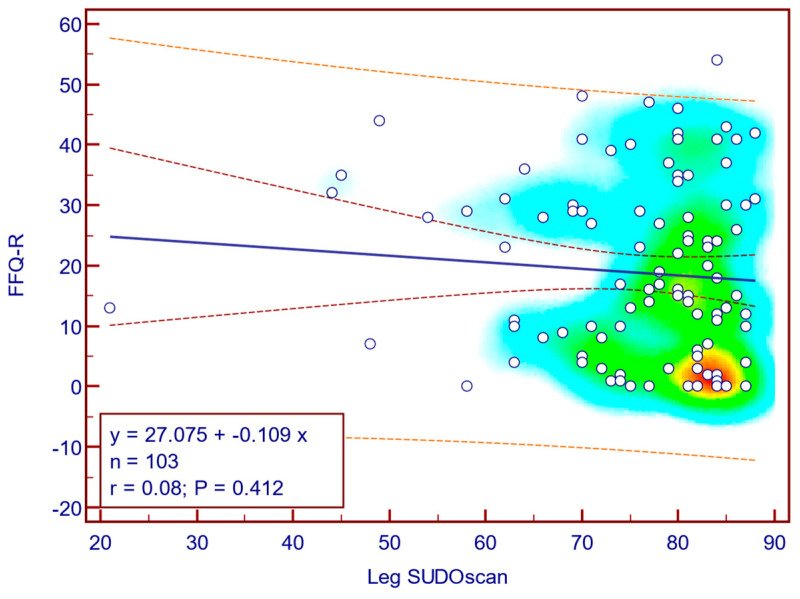
Correlation between FFQ-R score and leg SUDOSCAN values.

The FFQ-R score was weakly and not statistically significantly correlated with leg SUDOSCAN values (Spearman’s r = 0.08; *p* = 0.412; Figure 15), suggesting that there is no association between the severity of sudomotor dysfunction and fear of falling in patients with T2DM.

To evaluate the interaction between the MNSI questionnaire scores and MNSI objective evaluation scores in the prediction of the BBS score, a multivariate regression model was built, with the BBS score as the dependent variable and the MSNI questionnaire and MNSI objective scores as the independent variables. The model built using the two MNSI components explained 11.53% of the variations observed in the BBS score. The model demonstrated that the two MNSI components are independent predictors of balance impairment, as evaluated using the BBS score (Table 3).

To evaluate the interaction between the MNSI questionnaire scores and MNSI objective evaluation scores in the prediction of the TUG results, a multivariate regression model was built, with the TUG as the dependent variable and the MSNI questionnaire and MNSI objective scores as the independent variables. The model built using the two MNSI components explained 5.47% of the variations observed in the TUG value. The model demonstrated that only the MNSI questionnaire score is an independent predictor for TUG value alterations (Table 4).

To evaluate the interaction between the MNSI questionnaire scores and MNSI objective evaluation scores in the prediction of the FES-I score, a multivariate regression model was built, with the FES-I as the dependent variable and the MSNI questionnaire and MNSI objective scores as the independent variables. The model built using the two MNSI components explained 17.03% of the variations observed in the FES-I score. The model demonstrated that only the MNSI questionnaire score is an independent predictor for TUG value alterations (Table 5).

To evaluate the interaction between the MNSI questionnaire scores and MNSI objective evaluation score in the prediction of the FFQ-R score, a multivariate regression model was built, with the FFQ-R as the dependent variable and the MSNI questionnaire and MNSI objective scores as the independent variables. The model built using the two MNSI components explained 22.33% of the variations observed in the FFQ-R score. The model demonstrated that both MNSI questionnaire and MNSI objective scores are independent predictors for FFQ-R score changes (Table 6).

## 4. Discussion

### 4.1. Findings and Interpretation

Peripheral sensory and motor nerve damage associated with diabetic neuropathy affects up to half of all individuals with diabetes and constitutes an independent risk factor for falls. Diabetes contributes to the deterioration of muscle strength, a condition that worsens with the progression of diabetic neuropathy and leads to altered gait biomechanics, impaired balance, and an increased risk of falls [12]. Also, an accelerated decline in muscle strength has been observed in patients with symptomatic neuropathy. Similarly, a rapid loss of muscle mass is evident in the feet and lower legs [13]. Sensory feedback from the feet during walking is essential for activating the muscles involved in lower limb stabilization and balance control. Accurate sensory input from the feet is fundamental for the neuromuscular activation required to stabilize the lower limbs and maintain postural control during gait. In diabetic neuropathy, the absence of this feedback disrupts gait biomechanics, compromises balance, and heightens fall risk [14]. Changes in gait characteristics, such as slower walking speed, limited joint range of motion, and reduced joint activity, have been reported to occur even before the clinical manifestation of diabetic neuropathy [15]. It is worth noting that, as suggested by the study’s results, these patients with neuropathy, who exhibit an increased fear of falling and reduced confidence in performing routine movements, may consequently experience a decline in physical activity. As physical activity is a key component in T2DM management, neuropathy may indirectly exacerbate metabolic dysregulation. Metabolic imbalance is a central element in the progression and prognosis of neuropathy, potentially generating a “vicious cycle” between these components.

In contrast to the consistent associations observed between MNSI scores and both performance-based and psychological measures of balance, sudomotor dysfunction assessed by SUDOSCAN was not associated with any of the balance or fear-of-falling outcomes. Several psychological and methodological explanations are plausible. First, postural stability and gait predominantly rely on large myelinated sensory fibers conveying proprioceptive input, motor nerve function, and central integration, whereas SUDOSCAN mainly assesses small unmyelinated fibers in the glabrous skin. Sudomotor impairment may therefore capture a different domain of neuropathy than the one most directly responsible for balance deficits. Secondly, in an elderly cohort with long-standing T2DM, SUDOSCAN may exhibit floor or ceiling effects, limiting sensitivity to within-group variability relevant for balance. Finally, balance and fear of falling are highly multifactorial, depending not only on peripheral nerve function but also on sarcopenia, vision, vestibular function, polypharmacy, and psychological factors; this complexity may dilute any isolated effect of sudomotor dysfunction on the measured outcomes.

### 4.2. Strengths and Weaknesses of the Study

A major strength of the study is that it represents the first evaluation of this kind in the analyzed population. The study is particularly valuable given that the population may possess distinct socio-economic and cultural characteristics. Neuropathy was evaluated in the study using both the standard MNSI method and assessments of sudomotor dysfunction. Moreover, this is the first study to evaluate whether sudomotor dysfunction is associated with balance impairments.

An important limitation is the imbalance between the groups with and without neuropathy. Although the total sample size was prospectively estimated and achieved to ensure 80% power to detect a clinically relevant difference in BBS scores, the consecutive-case recruitment strategy resulted in a higher-than-expected prevalence of neuropathy in this outpatient cohort. Consequently, the subgroup without neuropathy is smaller, making its estimates more susceptible to random variation and outliers, thus reducing the precision and statistical power for between-group comparisons. To mitigate this limitation, we also used multivariable regression models including the whole cohort, in which neuropathy severity (MNSI components) was entered as a continuous predictor. Nonetheless, the unbalanced case–control ratio should be considered when interpreting the magnitude of between-group differences.

### 4.3. Differences Between Other Similar Studies

A study involving 62 participants—32 with T2DM (13 with neuropathy and 19 without) and 30 without T2DM—assessed balance under four conditions: stable vs. unstable platform and eyes open vs. eyes closed. An electromagnetic sensor system placed at the C7 vertebra was used to measure trunk displacement in the anterior–posterior and medial–lateral directions. Functional strength and mobility were measured via the Five-Times-Sit-to-Stand Test (FTSST), Berg Balance Scale (BBS), and Timed Up & Go (TUG) Test. Patients with T2DM, whether they have been clinically diagnosed with peripheral neuropathy or not, display impairments in postural control and functional strength when compared to non-diabetic healthy peers. The presence of neuropathy did not always add measurable additional deficits for all balance or mobility outcomes in this study [16].

Another study revealed that individuals with T2DM and associated peripheral neuropathy exhibit significantly poorer balance, gait performance, functional mobility, and functionality. Additionally, they have a higher occurrence of falls compared to those without T2DM. These findings underscore the importance of early assessment and intervention to address these impairments in the T2DM population [17].

Another study examined the relationship between balance confidence (a psychological factor) and fall risk in people with diabetic neuropathy to compare how psychological vs. functional parameters are associated with fall risk. As demonstrated by our study as well, psychological factors—specifically, confidence in one’s balance—have been shown to play a key role in determining fall risk among people with diabetic neuropathy. Interventions aiming to improve balance confidence (not just physical exercise) may be valuable in reducing fall risk in this population [18].

### 4.4. Relevance of the Findings

Defining a high-risk fall patient cluster may support personalized intervention strategies, such as targeted rehabilitation to enhance postural control in patients with neuropathy. Our findings show consistent associations between neuropathy burden and both objective and subjective measures of balance, supporting integration of systematic neuropathy and risk of falls screening into routine care for elderly adults with T2DM. In clinical practice, patients with T2DM and DN might benefit from structured, multifactorial interventions, including lower-limb and core strengthening, balance and gait training, tailored footwear and orthoses, home hazard assessment and reduction, and education programs focused on improving balance confidence and safe mobility. These rehabilitation strategies should be implemented alongside optimal metabolic control, cardiovascular risk management, and neuropathy-directed pharmacological treatment, aiming to reduce falls, preserve functional independence, and break the vicious cycle between neuropathy, fear of falling, and inactivity.

## 5. Conclusions

In this cohort of elderly adults with T2DM, the presence of DN was associated with impaired balance and increased fear of falling. Within the T2DM population, patients with neuropathy had lower BBS scores and higher FES-I and FFQ-R scores than those without neuropathy, indicating both objective and perceived balance deficits. Neuropathy burden, particularly patient-reported symptoms and signs captured by the MNSI questionnaire and examination components, independently predicted worse balance performance and greater fear of falling. By contrast, sudomotor dysfunction assessed by SUDOSCAN was not associated with balance or fear-of-falling measures, suggesting that small-fiber autonomic impairment alone does not sufficiently explain fall-related functional limitations in this population.

These findings highlight the need to systematically screen elderly patients with T2DM for neuropathy and fall risk and to implement targeted, multidisciplinary interventions. Comprehensive programs combining balance and strength training, gait and mobility exercises, optimization of footwear, review of medications that may increase the risk of falls, and education to enhance balance confidence should be prioritized for patients with T2DM and DN. Such interventions, together with optimal glycemic control and neuropathy management, may reduce falls, preserve independence and potentially attenuate the progression of neuropathy by supporting the maintenance of physical activity.

## Figures and Tables

**Table 1:** Patients’ baseline characteristics.

Parameter	Value
Age (years) ^a^	70.9 ± 5.9
Men ^b^	53 (42.7%)
Urban residence ^b^	69 (55.6%)
BMI (kg/m^2^) ^a^	31.8 ± 4.4
Diabetes duration (years) ^c^	9 [5–14.5]
HbA1c (percentage points) ^c^	7.3 [6.5–8.15]
Metformin usage ^b^	107 (86.3%)
Sulphonylurea usage ^b^	76 (61.3%)
DPP4i usage ^b^	24 (19.4%)
GLP1-RA usage ^b^	38 (30.6%)
SGLT2i usage ^b^	11 (8.9%)

^a^ Continuous variable with Gaussian distribution. Results are presented as average ± standard deviation. ^b^ Categorical variable. Results are presented as absolute frequencies and (percentage of the group’s total). ^c^ Continuous variable with non-parametric distribution. Results are presented as median and [interquartile range].

**Table 2:** Comparison of balance parameters in patients with vs. without neuropathy.

	Without Neuropathy (n = 15)	With Neuropathy (n = 109)	*p*-Value
FES-I (points)	21.0 [16.75–23.75]	31.0 [22.0–40.25]	0.001 *
FFQ-R (points)	5.0 [0.75–11.75]	23.0 [7.75–32.5]	0.006 *
BBS (points)	46.0 [41.25–54.75]	42.0 [40.0–44.0]	0.029 *
TUG (seconds)	11.25 [10.07–12.65]	11.8 [11.0–14.25]	0.699

Variables with nonparametric distributions. Results are presented as medians and [interquartile range]. *p*-value was calculated using Mann–Whitney U test. * Differences are significant at *α* = 0.05 significance threshold.

**Table 3:** Multivariate regression analysis for the impact of MNSI components on the BBS score.

Independent Variables	Coefficient	Std. Error	95% CI	*t*	*p*
**(Constant)**	**50.65**	**2.41**	**45.87 to 55.43**	**20.9**	**<0.0001**
**MNSI Questionnaire**	**−0.74**	**0.36**	**−1.47 to −0.027**	**−2.1**	**0.0420**
**MNSI Objective**	**−1.10**	**0.48**	**−2.05 to −0.14**	**−2.3**	**0.0237**

**Table 4:** Multivariate regression analysis for the impact of MNSI components on the TUG value.

Independent Variables	Coefficient	Std. Error	95% CI	*t*	*p*
**(Constant)**	**11.32**	**1.16**	**9.03 to 13.62**	**9.75**	**<0.0001**
**MNSI Questionnaire**	**0.43**	**0.17**	**0.09 to 0.78**	**2.48**	**0.0143**
**MNSI Objective**	**−0.038**	**0.23**	**−0.49 to 0.41**	**−0.16**	**0.8684**

**Table 5:** Multivariate regression analysis for the impact of MNSI components on the FES-I score.

Independent Variables	Coefficient	Std. Error	95% CI	*t*	*p*
**(Constant)**	**19.52**	**2.81**	**13.95 to 25.09**	**6.93**	**<0.0001**
**MNSI Questionnaire**	**1.66**	**0.42**	**0.82 to 2.50**	**3.90**	**0.0002**
**MNSI Objective**	**0.90**	**0.56**	**−0.20 to 2.01**	**1.60**	**0.1107**

**Table 6:** Multivariate regression analysis for the impact of MNSI components on the FFQ-R score.

Independent Variables	Coefficient	Std. Error	95% CI	*t*	*p*
**(Constant)**	**2.12**	**3.76**	**−5.33 to 9.58**	**0.56**	**0.5733**
**MNSI Questionnaire**	**2.32**	**0.57**	**1.19 to 3.44**	**4.07**	**0.001**
**MNSI Objective**	**1.71**	**0.75**	**0.22 to 3.19**	**2.27**	**0.0247**

## Data Availability

The data presented in this study are available on request from the corresponding author. The data are not publicly available due to local privacy and data protection regulations.

## Abbreviations

The following abbreviations are used in this manuscript:

DN	Diabetic neuropathy
T2DM	Type 2 diabetes mellitus
MNSI	Michigan Neuropathy Screening Instrument
BBS	Berg Balance Scale
TUG	Timed Up and Go
FES-I	Falls Efficacy Scale—International
FFQ-R	Fear of Falling Questionnaire—Revised
T1DM	Type 1 diabetes mellitus
HbA1c	Hemoglobin A1c
LDLc	Low-density lipoprotein cholesterol
HDLc	High-density lipoprotein cholesterol
AST	Aspartate aminotransferase
ALT	Alanine aminotransferase
hsCRP	High-sensitivity C-reactive protein
FTSST	Five-Times-Sit-to-Stand Test
